# Integrated Display and Simulation for Automatic Dependent Surveillance–Broadcast and Traffic Collision Avoidance System Data Fusion

**DOI:** 10.3390/s17112611

**Published:** 2017-11-13

**Authors:** Yanran Wang, Gang Xiao, Zhouyun Dai

**Affiliations:** School of Aeronautics and Astronautics, Shanghai Jiao Tong University, 800 Dongchuan Road, Minhang District, Shanghai 200240, China; yrwang501@163.com (Y.W.); m18792833886@163.com (Z.D.)

**Keywords:** ADS-B, TCAS, integrated display, data fusion, airspace surveillance

## Abstract

Automatic Dependent Surveillance–Broadcast (ADS-B) is the direction of airspace surveillance development. Research analyzing the benefits of Traffic Collision Avoidance System (TCAS) and ADS-B data fusion is almost absent. The paper proposes an ADS-B minimum system from ADS-B In and ADS-B Out. In ADS-B In, a fusion model with a variable sampling Variational Bayesian-Interacting Multiple Model (VSVB-IMM) algorithm is proposed for integrated display and an airspace traffic situation display is developed by using ADS-B information. ADS-B Out includes ADS-B Out transmission based on a simulator platform and an Unmanned Aerial Vehicle (UAV) platform. This paper describes the overall implementation of ADS-B minimum system, including theoretical model design, experimental simulation verification, engineering implementation, results analysis, etc. Simulation and implementation results show that the fused system has better performance than each independent subsystem and it can work well in engineering applications.

## 1. Introduction

ADS-B [1,2] is defined by the U.S. Federal Aviation Administration (FAA) as the foundation of free flight. It is an applicable and accurate airspace surveillance technology. It can be used in traffic collision avoidance, surveillance, auxiliary approach and may have a great effect on all those aspects. TCAS [3,4] is an airborne traffic alarm and collision avoidance system which is independent of air traffic control on the ground.

The fusion of ADS-B and TCAS data can improve the prediction accuracy of TCAS systems [5], increase the rate of true alarms and decrease the rate of false and missed alarms. There are many benefits for airspace alarm accuracy and flight safety by combining with TCAS II and ADS-B system. Even if essential information of a single system is missing, the system will work and not be affected too much. The application of these technologies is closely related to the next generation of air traffic systems, which are expected to have many benefits for the manufacture and use of civil aircraft.

Regarding the ADS-B system aspects, Kunzi [3] analyzed the benefits of ADS-B in general aviation and pointed out that ADS-B will be the basis of the future surveillance system. Wang [6] designed and implemented a complete mini ADS-B air traffic control system based on five aspects which are monitoring data displaying, flight data displaying, ADS-B message processing, alarm computing and equipment running status monitoring. McCallie [7] at the Air Force Institute of Technology performed a safety analysis of the next generation of air traffic transportation. Mueller [8] proposed Aircraft ADS-B Intent Verification based on a Kalman Tracking Filter in order to improve the accuracy of ADS-B data transmission and to reduce the data error rate. Peng [9] proposed the CPR algorithm for 1090ES ADS-B system encoding and decoding to improve transmission efficiency. Purton [10] solved some of the uncertainties in ADS-B. The procedural remedies are proposed for technical problems. Purton also focused on technical solutions for the vulnerabilities and threats in the transmission and computation.

In the aspects of ADS-B and TCAS fusion, Ni [11] studied a ADS-B and TCAS II system fusion algorithm. The Kalman filter is used and the data fusion is carried out according to the optimal information fusion criterion with the minimum linear variance. Xu [12] proposed the local trajectory estimation, optimized ADS-B local trajectory estimation of TCAS and the optimized fusion trajectory estimations based on the data fusion algorithm of the Current Statistical (CS) model, however, it is not appropriate for civil aircraft when cruising.

Many scholars have proposed or improved the Interacting Multiple Model (IMM) and Variational Bayesian (VB) algorithm. A Sense-and-Avoid (SAA) system and IMM model is used to estimate the state of intruders by Ramsamy [13]. Sabatini and Chen [14,15] introduced a SAA system. An extended Kalman Filter and common IMM [16] algorithm were used to estimate the intruder’s state vector. The IMM tracking models were focused on by the F.A.A. and the Thales Company. Constant Velocity (CV), Constant Acceleration (CA) and Constant Turning (CT) models are designed for surface tracking [17] and finally IMM was used to improve the accuracy of tracking. IMM has the characteristic of being adaptive, and can effectively adjust the probability of each model. However, few methods which are practical for real engineering issues. Variational Bayesian (VB) methods have been developed for a wide range of models to perform approximate posterior inference at low computational cost in comparison with the sampling methods (for a review of VB methods, see, e.g., [18,19]). The VB method was utilized by Sarkka [20] to propose an adaptive Kalman filtering method which is based on forming a separable variational approximation to the joint posterior distribution of states and noise parameters on each time step separately.

Due to the fact the small deviations in the previous simulation is not sufficient to prove the effectiveness of the fusion benefit, we built a fusion system based on a combined ADS-B physical system and TCAS simulation. The ADS-B minimum system is defined as a system with ADS-B equipment as the main part. It is in a semi-physical simulation environment which has some minor differences with the real system. An ADS-B and TCAS data fusion model based on an adaptive sampling period VB-IMM algorithm is proposed for integrated display from a theoretical model design, experimental simulation verification, engineering implementing the three parts in ADS-B In. An airspace traffic situation display is also presented. In ADS-B Out, some experimental verification processes include ADS-B Out data transmission based on a simulation cockpit platform and ADS-B Out data transmission based on an UAV platform.

The paper is organized as follows: Section 2 presents the design of ADS-B minimum system from two aspects of ADS-B In and Out including mathematical formulations and engineering implementation methods. Section 3 presents flight simulation and engineering implementation results of our ADS-B minimum system. Section 4 presents our conclusions and discusses future challenges and perspectives.

## 2. ADS-B Minimum System Design

**Figure 1:**
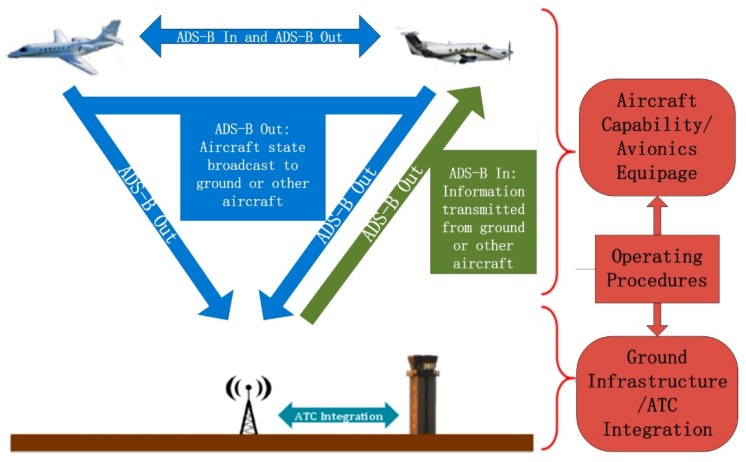
Automatic Dependent Surveillance–Broadcast (ADS-B) data transmission.

**Figure 2:**
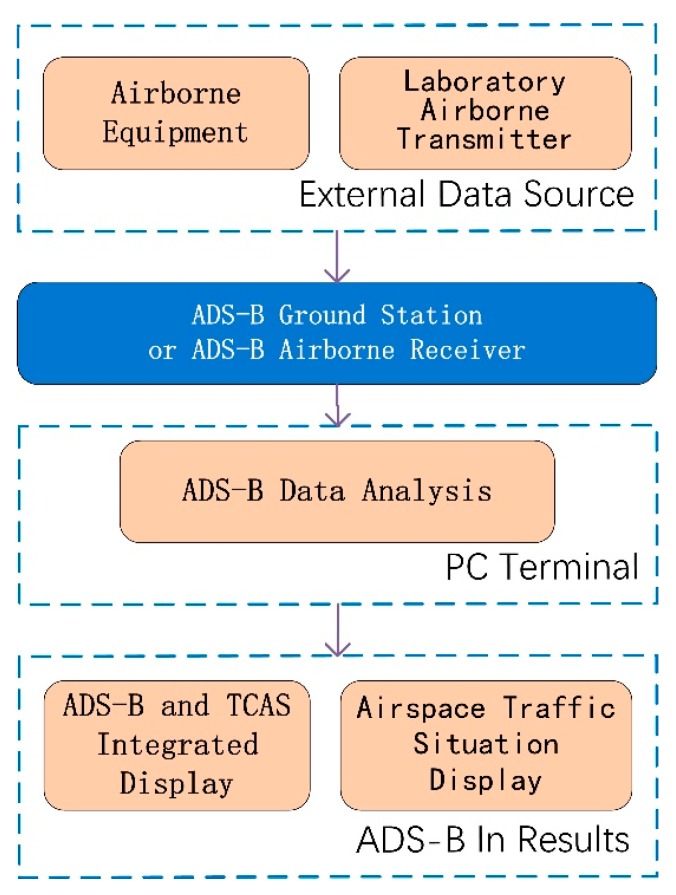
ADS-B In minimum system design.

The ADS-B minimum system design is shown from the two aspects of ADS-B In and Out. The processes of ADS-B In and Out are shown in Figure 1. The main theoretical design content of the ADS-B minimum system is a fusion model with a variable sampling VB-IMM (VSVB-IMM) algorithm. The part of engineering design and experimental verification includes airspace traffic situation display, ADS-B Out data transmission based on a simulation cockpit and UAV platform. The ADS-B minimum system design is shown in Figure 2 and Figure 5.

### 2.1. ADS-B In Minimum System Design

The steps of ADS-B In is that the broadcast data are received at an ADS-B station or ADS-B airborne equipment from 1090 MHz frequency and then the data is sent to the PC terminal through a serial port or network port for data processing. The integrated display and airspace traffic situation display is shown as the results of ADS-B In. The program of ADS-B In is shown in Figure 2.

#### 2.1.1. ADS-B and TCAS Integrated Display Development

**Figure 3:**
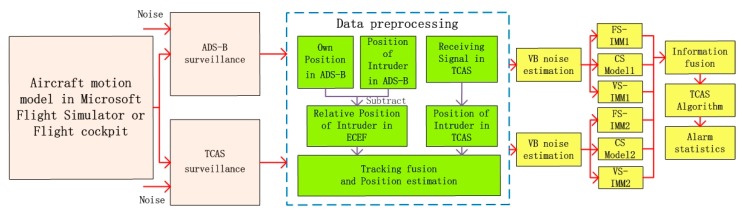
System frame diagram.

A VB-IMM algorithm was given by Dai [5] for maneuvering target tracking and estimating unknown noise variance. The 3-dimensional trajectory is generated by an aircraft movement model or flight simulation cockpit and IMM is utilized for filtering. The time-varying noise is estimated by the VB method, which is the basis for failure prediction and adjustment of the sampling period. The CV, CA and CS filter model is built in parallel in the IMM algorithm [21]. The state can be estimated by the filter weighting of different motion models. The local optimal value is obtained from variable sampling IMM (VS-IMM), fixed sampling IMM (FS-IMM) and the CS model algorithm. The global optimal value is obtained from the local optimal value following the optimal information fusion criterion and is used as input of the TCAS subsystem for obtaining the value of Tau (time until the closest point of approach) between aircraft. False alarms and leak alarms are counted for statistical analysis. The system frame diagram is shown in Figure 3.

1. Data preprocessing

(1) Coordinate transformation

The ADS-B coordinate in WGS-84 and TCAS II data is the relative position. The coordinates of ADS-B and TCAS are transformed into a unified fusion coordinate system which is the “Earth-Centered, Earth-Fixed (ECEF)” coordinate system.

(2) Time synchronization

Although the update rate for ADS-B reports and TCAS II data is 1 s, the system delay will result in inconsistencies with data acquisition. Therefore, the data must be synchronized before data fusion. Since the ADS-B is based on GNSS, the ADS-B time base is used as a benchmark, so the measured values of TCAS II at time $t_{j}$ are synchronized to the common processing time $t_{i}$ of ADS-B which is shown in Equation (1):

(1) $$Z_{TCAS\;II}\left(t_{i}\right)=Z_{TCAS\;II}\left(t_{j}\right)+v\times \left(t_{i}-t_{j}\right)$$

where $Z_{TCAS\;II}\left(t_{i}\right)$ is the measured values of TCAS II in time $t_{i}$. v is the velocity of aircraft and the $v\times \left(t_{i}-t_{j}\right)$ is a correction item.

2. Markov jump linear system and IMM model

The Markov jump linear system [22,23] is shown in Equations (2)–(5):

(2) $$MX\left(k+1\right)=\emptyset_{j}\left(k\right)X\left(k\right)+\omega \left(k\right)$$

(3) $$Z\left(k\right)=H_{j}X\left(k\right)+v_{j}\left(k\right)$$

(4) ω(k+1)=cω(k)+ξ(k)

(5) $$v_{j}\left(k+1\right)=d_{j}v_{j}\left(k\right)+\eta_{j}\left(k\right)$$

where M is the singular square matrix which means detM=0. The system is regular which means det (λM−∅) is not constantly 0. The state vector X(k) is an n-dimensional vector, the observation process Z(k) is an m-dimensional vector and the subscript jϵS={1,2,…,s} denotes the model. The matrix functions $\emptyset_{j}\left(k\right)$ and $H_{j}\left(k\right)$ are known. The vector ξ(k) is a zero-mean white Gaussian process noise and the $\eta_{j}\left(k\right)$ is an independent Gaussian measurement noise with zero-mean and variance to be estimated.

Applying the augmented state method to convert the colored process noise ω(k) into part of the system state. After the original system state component is expanded, the new system is defined as Equations (6) and (7):

(6) $$\bar{X}\left(k+1\right)=\bar{\emptyset }\left(k\right)\bar{X}\left(k\right)+\bar{\Gamma }\xi \left(k\right)$$

(7) $$Z\left(k\right)=\bar{H_{j}}\bar{X}\left(k\right)+\gamma_{j}\left(k\right)$$

where $\bar{X}\left(k\right)=\left[\begin{array}{c} X_{1}\left(k\right)\\ \omega \left(k\right) \end{array}\right]$, $\bar{\emptyset }=\left[\begin{array}{cc} {L_{1}}^{-1}T_{1} & {L_{1}}^{-1}\Gamma_{1}\\ 0 & c \end{array}\right]$, $\bar{\Gamma }=\left[\begin{array}{c} 0\\ 1 \end{array}\right]$. The colored process noise ω(k) converts to the white noise ξ(k). It exists orthogonal matrix P and *Q* to satisfy the equation $\mathrm{PMQ}=\left[\begin{array}{cc} L_{1} & 0\\ L_{2} & 0 \end{array}\right]$. $L_{1}$ is lower non-singular triangular matrix. $\bar{H_{j}}=H_{j}\left(\bar{\emptyset }-d_{j}I\right)$, $\gamma_{j}\left(k\right)=H_{j}\bar{\Gamma }\xi \left(k\right)+\eta \left(k\right).$ The colored process noise $v_{j}\left(k\right)$ converts to the white noise $\gamma_{j}\left(k\right)$.

The paper describes the IMM algorithm with three models, including CV, CA and CS model. X(k) is the state vector of target and it is a 3-dimensional vector consisting of position, velocity and acceleration which is described in Equation (8):

(8) $$X\left(k\right)=\left[x\;\dot{x}\;\ddot{x}\right]$$

Zhou [24] proposed the CS model and processed the noise of maneuvering target acceleration with a Rayleigh distribution. The CS model is described in Equations (9)–(12):

(9) $$X\left(k+1\right)=\emptyset \left(k\right)X\left(k\right)+T\left(k\right)\bar{a}+\omega \left(k\right)$$

(10) $$Z\left(k\right)=H_{j}X\left(k\right)+v_{j}\left(k\right)$$

(11) $$\emptyset \left(k\right)=\left[\begin{array}{ccc} 1 & T & \frac{\left(-1+aT+e^{-aT}\right)}{a^{2}}\\ 0 & 1 & \frac{\left(1-e^{-aT}\right)}{a}\\ 0 & 0 & e^{-aT} \end{array}\right],\;T\left(k\right)=\left[\begin{array}{c} \frac{\left(-T+\frac{aT^{2}}{2}+\frac{\left(1-e^{-aT}\right)}{a}\right)}{a}\\ T-\frac{\left(1-e^{-aT}\right)}{a}\\ 1-e^{-aT} \end{array}\right]$$

(12) $$Q\left(k\right)=2a{\sigma_{a}}^{2}Q$$

The process noise matrix Q(k). Q is a constant matrix. ${\sigma_{a}}^{2}$ is the maneuvering acceleration variance. Maneuvering acceleration variance can be obtained in Equation (13):

(13) $${\sigma_{a}}^{2}=\{\begin{array}{l} \frac{4-\pi }{\pi }\left(a_{max}-\hat{a}\left(k|k-1\right)\right),\;if\;a\geq 0\\ \frac{4-\pi }{\pi }\left(\hat{a}\left(k|k-1\right)-a_{-max}\right),\;if\;a<0 \end{array}$$

where $a_{max}$ and $a_{-max}$ are the upper and lower acceleration limits and the acceleration estimation is $\hat{a}\left(k|k-1\right)$. The closer the real value of acceleration is to $a_{max}$, the smaller the ${\sigma_{a}}^{2}$ is. The closer the real value of acceleration is to zero, the larger the ${\sigma_{a}}^{2}$ is. The value of ${\sigma_{a}}^{2}$ affects the process noise Q(k) which decides the tracking accuracy of maneuvering targets. The problem is how to make the system have a constant speed or a small and large degree number of maneuvers track accurately as much as possible and scholars have put forward ideas for this:

(14) $$f\;\left(x,y,\sigma \right)=1-e^{-\frac{{\left(x-y\right)}^{2}}{\mu \sigma^{2}}}$$

(15) $$Q^{\prime }\left(k\right)=Q\;\left(k\right)*f\;\left(x,y,\sigma \right)=2a{\sigma_{a}}^{2}Q*\left(1-e^{-\frac{{\left(x-y\right)}^{2}}{\mu \sigma^{2}}}\right)$$

Li [25] used Gaussian membership function of fuzzy control theory to improve the CS model and the Equations (14) and (15) show the details of the improved model. The terms x, y, $\sigma^{2}$ and μ are the input of position, the estimation of current position, the variance of innovation and a constant, respectively. The value |x−y| is big when there is a large maneuver, so f (x,y,σ)→1. Meanwhile Q(k) is small and the improved model $Q^{\prime }\left(k\right)$ is relatively small. On the contrary, a small maneuver causes f(x,y,σ)→0 and the value of Q(k) is large, so it can also guarantee $Q^{\prime }\left(k\right)$ is stable.

3. The formal algorithm flow

**Figure 4:**
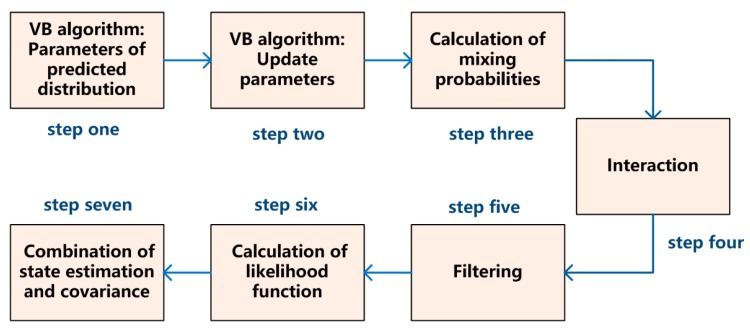
The formal algorithm flow.

The formal algorithm flow is shown in Figure 4. The unknown measuring noise variance is estimated by the VB algorithm [26] which is in step one and two. $m_{k}^{-}$ is the acceleration estimation and $P_{k}^{-}$ is the a priori state covariance. The main equation is shown as follows:

(16) $$m_{k}^{-}=\emptyset \left(k\right)m_{k-1}^{-}+T\left(k\right)m_{k-1}^{-}$$

(17) $$P_{k}^{-}=\emptyset \left(k\right)P_{k-1}\emptyset {\left(k\right)}^{T}+Q\left(k\right)$$

The estimation of noise variance is input into the IMM model. The process of IMM filtering [27] is shown in steps three, four and five.

In step three, the process of calculation of mixing probabilities is described in Equation (18), where $\bar{c_{j}}$ is a normalization factor:

(18) $$\mu_{i|j}\left(k-1|k-1\right)=\left(\frac{1}{\bar{c_{j}}}\right)p_{ij}\mu_{i}\left(k-1\right),\;\bar{c_{j}}=\underset{i}{\sum }p_{ij}\mu_{i}\left(k-1\right)$$

In step four, the system state is estimated which is calculated by a linear equation with ${\hat{x}}_{i}\left(k-1|k-1\right)$, $\mu_{i|j}\left(k-1|k-1\right)$, $P_{i}\left(k-1|k-1\right)$, etc.

In step five, the measurement noise variance is obtained by VB estimation rather than a predetermined constant in the common IMM algorithm. The state estimation and covariance matrix are obtained by running each filter.

In step six, the likelihood function is calculated by the residual measurement and covariance. Finally, in step seven, $\hat{x}\left(k|k\right)$ and $\hat{p}\left(k|k\right)$ are estimated by a weighted sum of the estimations from all filters.

4. Variable sampling period VB-IMM [28]

The sampling period of VB-IMM is shown in Table 1. The F.A.A. issued the problem Airworthiness Approval of Automatic Dependent Surveillance-Broadcast Out systems in 2011 [29]. The concept of Navigation Accuracy Category for Position (${\mathrm{NAC}}_{P}$) specifies the accuracy of the aircraft’s horizontal position information (latitude and longitude) transmitted from the aircraft’s avionics. EPU is the Estimated Position Uncertainty. The sampling period is determined by position variance and ${\mathrm{NAC}}_{P}$. The sampling period can increase appropriately when the variance estimated by the VB algorithm is relatively small, so the simulation sampling period is 1 s in the ${\mathrm{NAC}}_{P}$ 10 and 11 in Table 1. The sampling should be intensive when the estimated variance increases and meantime the intruder is closer. The sampling period in the simulation is 0.8 s in the ${\mathrm{NAC}}_{P}$ 9 and 0.6 s in the ${\mathrm{NAC}}_{P}$ 8, 7, 6. When the intruder is far away, the sampling period is restored to 1 s. The simulation sampling period is adaptive according to the value of ${\mathrm{NAC}}_{P}$.

5. Optimal Information Fusion Criterion

The unbiased estimations of L sensors, $\hat{x},\;i=1,\ldots ,L$. The estimation error covariance matrix $P_{ij},\;i=1,\ldots ,L$ is obtained. The fusion is performed according to a matrix weighted linear minimum variance criterion. There are two sensors (ADS-B and TCAS) in this paper, so L = 2. The process of fusion is given in Equations (19) and (20):

(19) $$\left[A_{1},\ldots ,A_{L}\right]=\left[A_{1},A_{2}\right]=\left[A_{TCAS},A_{ADS-B}\right]={\left(e^{T}P^{-1}e\right)}^{-1}e^{T}P^{-1}$$

(20) $${\hat{X}}_{f}=\overset{L}{\underset{i=1}{\sum }}A_{i}{\hat{x}}_{i}=A_{1}{\hat{x}}_{1}+A_{2}{\hat{x}}_{2}=A_{TCAS}{\hat{x}}_{TCAS}+A_{ADS-B}{\hat{x}}_{ADS-B}$$

where $A_{i}$ is an *n*-order square matrix, P is a block matrix with $P_{ij}$ as the (i,j) element and $P_{ij}=cov\;\left({\hat{x}}_{i},{\hat{x}}_{j}\right),\;i,j=1,2$. $e=\left[I_{n,\ldots ,}I_{n}\right]$, $I_{n}$ is an nth-order identity matrix. We can hypothesize that the ${\hat{x}}_{TCAS}$ and ${\hat{x}}_{ADS-B}$ are unbiased because they are obtained from the results of variable sampling VB-IMM, fixed sampling VB-IMM and the CS model algorithm.

#### 2.1.2. Airspace Traffic Situation Display Development

The source of data which is received by an ADS-B station or ADS-B airborne equipment is from the ADS-B signals of aircraft in the airspace. After decoding an ADS-B messages and transferring the data to the PC terminal, the flight information is obtained. The flight information includes longitude, latitude, height, ground speed, ground velocity direction, vertical velocity, etc. in plaintext. The flight information is displayed on the airspace traffic situation interface. The paper develops two airspace traffic situation interfaces according to different requirements for surveillance systems.

### 2.2. ADS-B Out Minimum System Design

**Figure 5:**
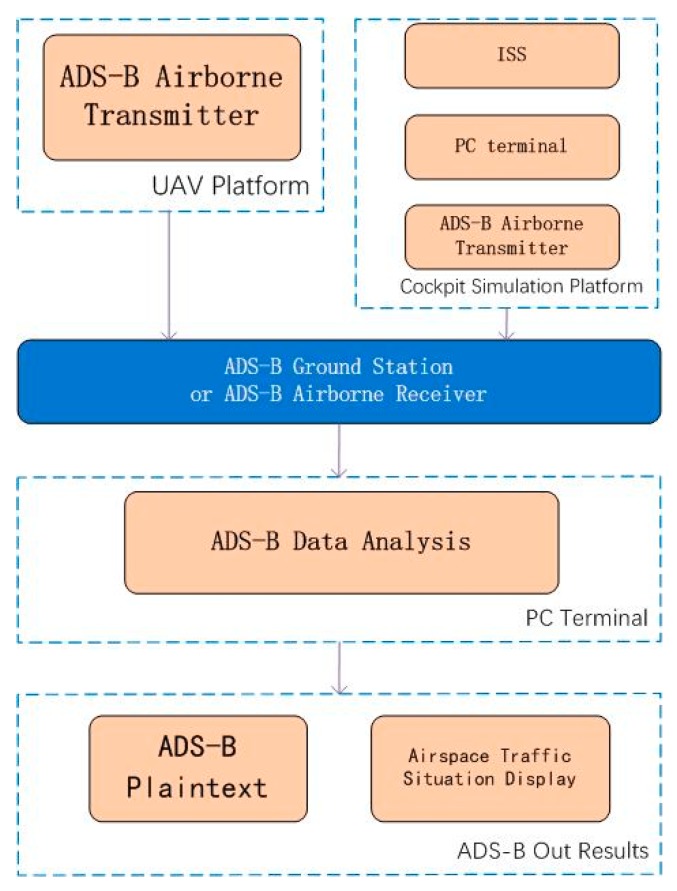
ADS-B Out minimum system design.

ADS-B Out is designed from two aspects. The first is the ADS-B Out data transmission based on a simulation cockpit platform. The second is ADS-B Out data transmission based on an UAV platform. The program of ADS-B Out is shown in Figure 5.

#### 2.2.1. ADS-B Out Data Transmission Based on a Simulation Cockpit Platform

The simulation flight information is generated by the simulation cockpit platform. The flight information of the aircraft are sent to a PC terminal through an Integrated Surveillance System (ISS). After it is processed and converted by the protocols in the PC terminal, data are sent to the ADS-B airborne equipment. Then they are transmitted to the airspace. The ADS-B data is received by the ADS-B station or ADS-B airborne equipment. After decoding, the flight information can be obtained.

#### 2.2.2. ADS-B Out Data Transmission Based on an UAV Platform

ADS-B airborne equipment is loaded on the UAV platform and it obtains the current flight information through the airborne equipment GPS and Beidou modules. The ADS-B signal is received by the ADS-B ground station or ADS-B airborne equipment, and the aircraft flight information is obtained after decoding. The UAV flight path is displayed and is compared with the flight path that is recorded by the positioning device of the UAV itself.

**Figure 6:**
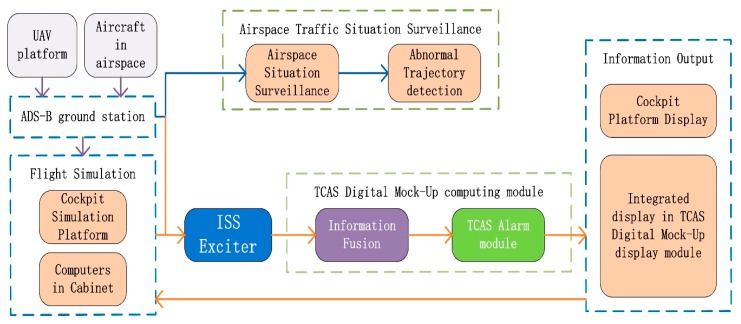
System design block diagram.

The main part of ADS-B minimum system is designed and shown in Figure 6. The three data sourcee of this system include the flight simulation module, aircraft in airspace and the experimental UAV platform. After decoding, the plaintext information can be used in two ways. The first is that the plaintext information is sent to the display terminal to surveil the airspace traffic situation and detect tany abnormal trajectory based on the recorded trajectory information or the flight recorder data. The second way is to send it to a Digital Mock-up Module to process the aircraft information including information estimation and fusion, then into TCAS logic, to carry out a collision avoidance solution. Finally, all this information is displayed on a TCAS Digital Mock-up and cockpit platform.

## 3. ADS-B Minimum System Implementation

### 3.1. ADS-B In Minimum System Implementation

#### 3.1.1. ADS-B and TCAS Integrated Display

This scheme is the core of the ADS-B minimum system. According to the previous analysis of the ADS-B In minimum system program, this part shows the results of a VB-IMM algorithm simulation and application of an integrated display with the ADS-B and TCAS fusion system.

##### Simulation Results of Fusion Model Based on VB-IMM Algorithm

The 3-dimensional trajectory is generated by an aircraft movement model or flight simulation cockpit. The IMM model is utilized for filtering. The VB method is used to estimate the time-varying noise. The false alarm, leak alarm statistics and the data fusion’s benefit analysis for the fused system are given to analyze the performance of system.

1. Flight path simulation

**Figure 7:**
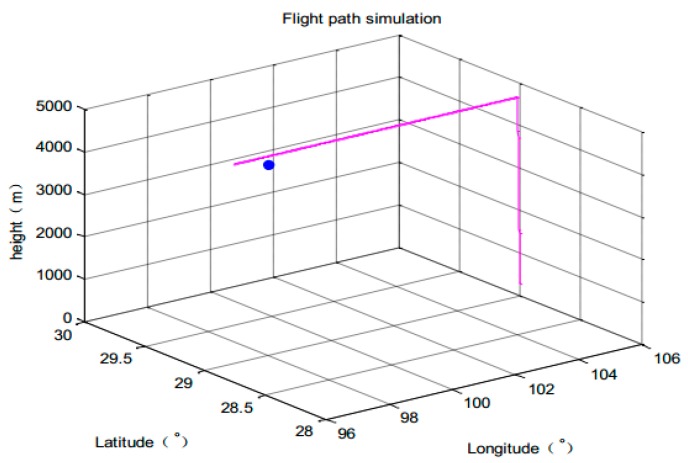
Aircraft trajectory.

The parameters of simulation system are set as follows: Flight experience 3000 s, sampling period T = 1 s. Flight position: 98°00′00″ E, 29°00′00″ N, 4502 m height. It climbs from 300 m height and then cruises at constant height. The initial position of the intruder: 106°00′00″ E, 29°00′00″ N, 300 m height. The observation noise variance of TCAS is 50 dB, ADS-B’s observation noise standard deviation is time-varying. The 3-dimensional trajectory is shown in Figure 7.

2. Variable sampling period VB-IMM (VSVB-IMM) and fixed sampling period VB-IMM (FSVB-IMM)

**Figure 8:**
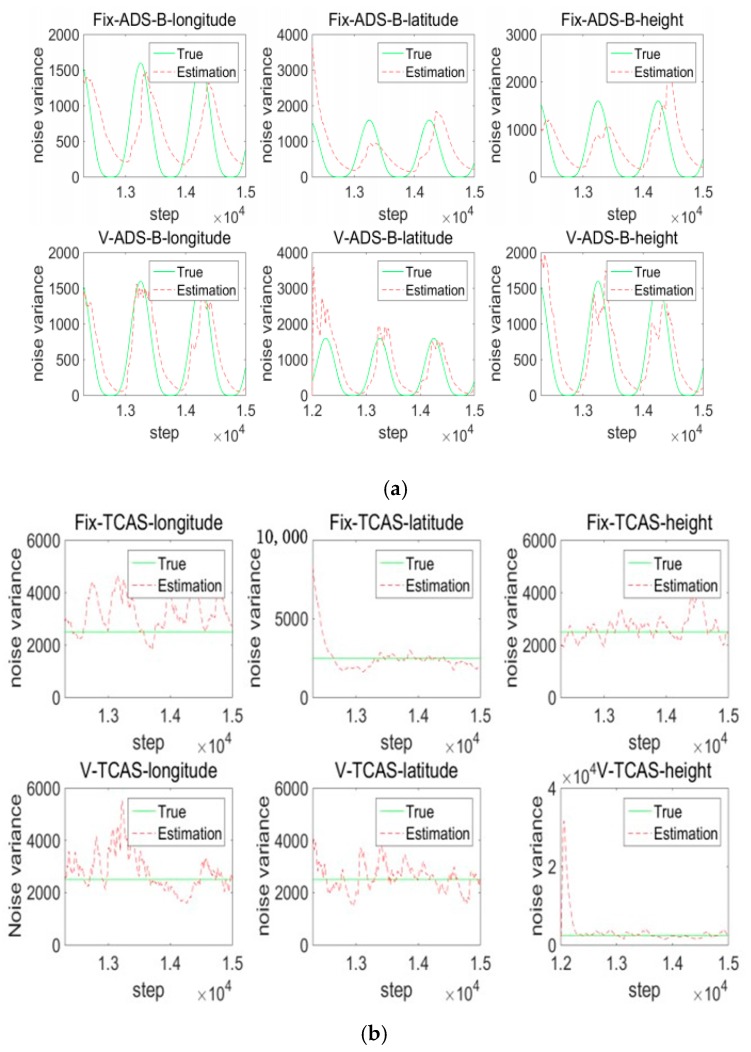
Noise estimation of system. (**a**) Noise estimation of ADS-B; (**b**) Noise estimation of Traffic Collision Avoidance System (TCAS).

Noise is estimated by a Variational Bayesian. The observation noise variance of TCAS is 50 dB and the ADS-B’s observation noise standard deviation is time-varying. In Figure 8, the variance of sinusoidal oscillation noise is 40 dB. The iteration is 30 in each cycle of this paper. Figure 8a,b are the online variance estimation of measurement noise for ADS-B and TCAS.

**Figure 9:**
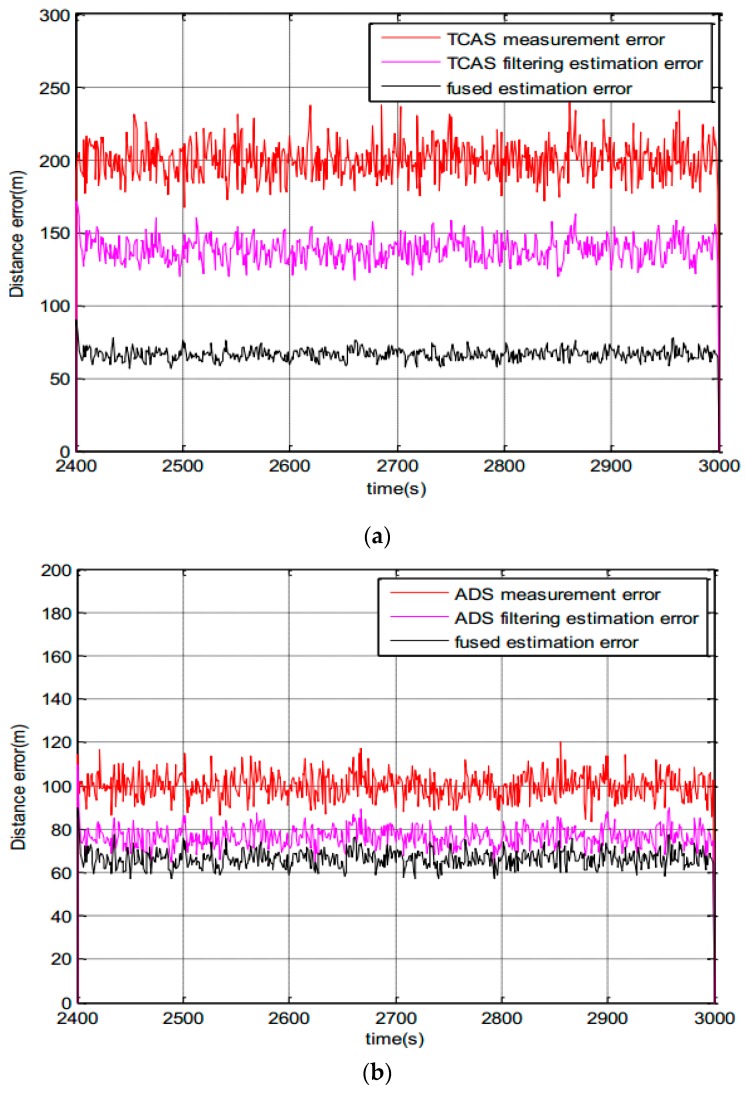
Measurement noise and fusion estimation noise. (**a**) Measurement noise and fusion estimation noise of TCAS; (**b**) measurement noise and fusion estimation noise of ADS-B.

The following is the statistical results of experiments. Combining Figure 9a with Figure 9b for analysis, we see the fused system’s estimation error is smaller than the TCAS and ADS-B subsystem’s estimation error. The fused track information is better than that of any subsystem. The fusion system is in a semi-physical simulation environment. A minimum system which combines the ADS-B physical system and TCAS simulation is built. The ADS-B measured values are obtained from an ADS-B Out experiment based on the simulator cockpit platform, which are sent to the air by the ADS-B airborne equipment, and received by an ADS-B ground station. Then the ADS-B measured noise is obtained from the ADS-B truth values and measured values.

3. Statistics of root mean squared error

**Figure 10:**
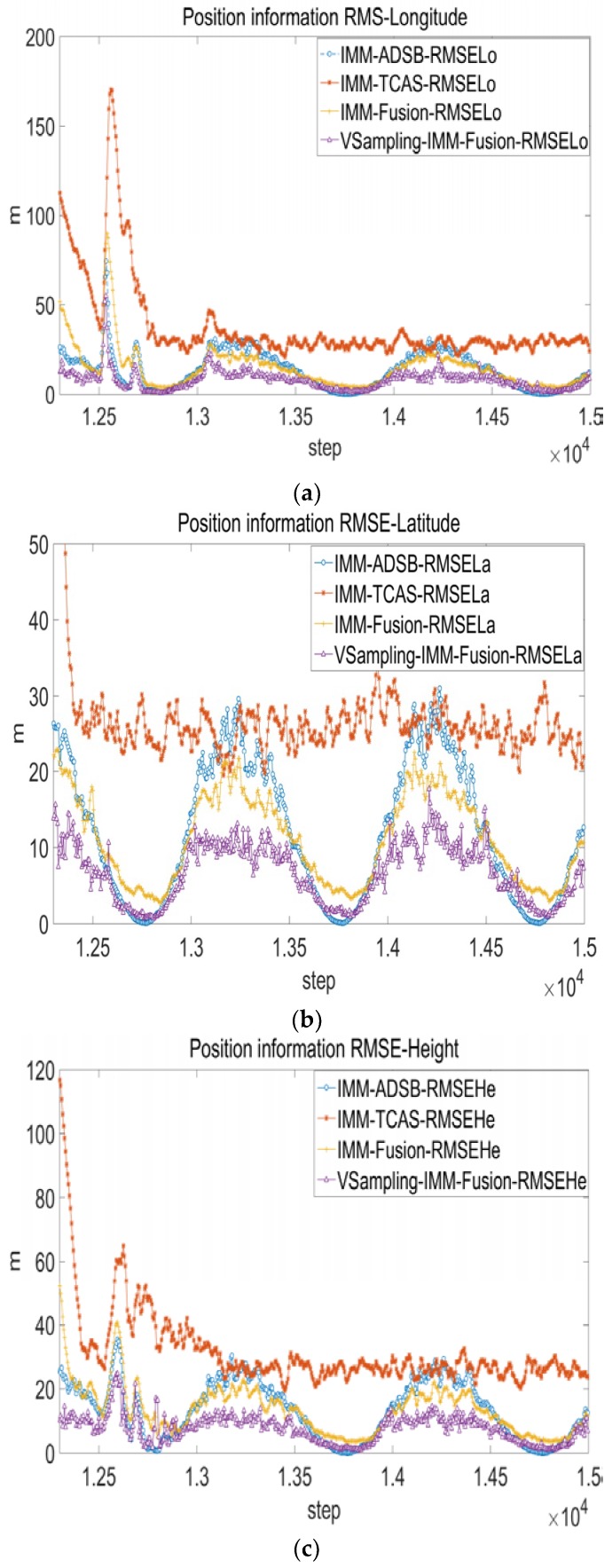
Root mean squared error of statistics. (**a**) Root mean squared error-Longitude; (**b**) Root mean squared error-Latitude; (**c**) Root mean squared error-Height.

The RMSE is calculated as Equation (21):

(21) $${\mathrm{RMSE}}_{k}={\left[\frac{1}{M}\overset{M}{\underset{i}{\sum }}{\left(x_{k}^{\left(i\right)}-\hat{x_{k}^{\left(i\right)}}\right)}^{2}\right]}^{\frac{1}{2}},\;k=1,2,\ldots ,\mathrm{step}$$

where M is the number of Monte Carlo iterations, the parameter k is the simulation step. The statistical result of 60 Monte Carlo experiments is shown in Figure 10.

The RMSE of fusion system is smaller than TCAS and ADS-B subsystem. The VSVB-IMM is superior to FSVB-IMM as purple line with triangles is the smallest most of the time.

4. TCAS CPA calculation

**Figure 11:**
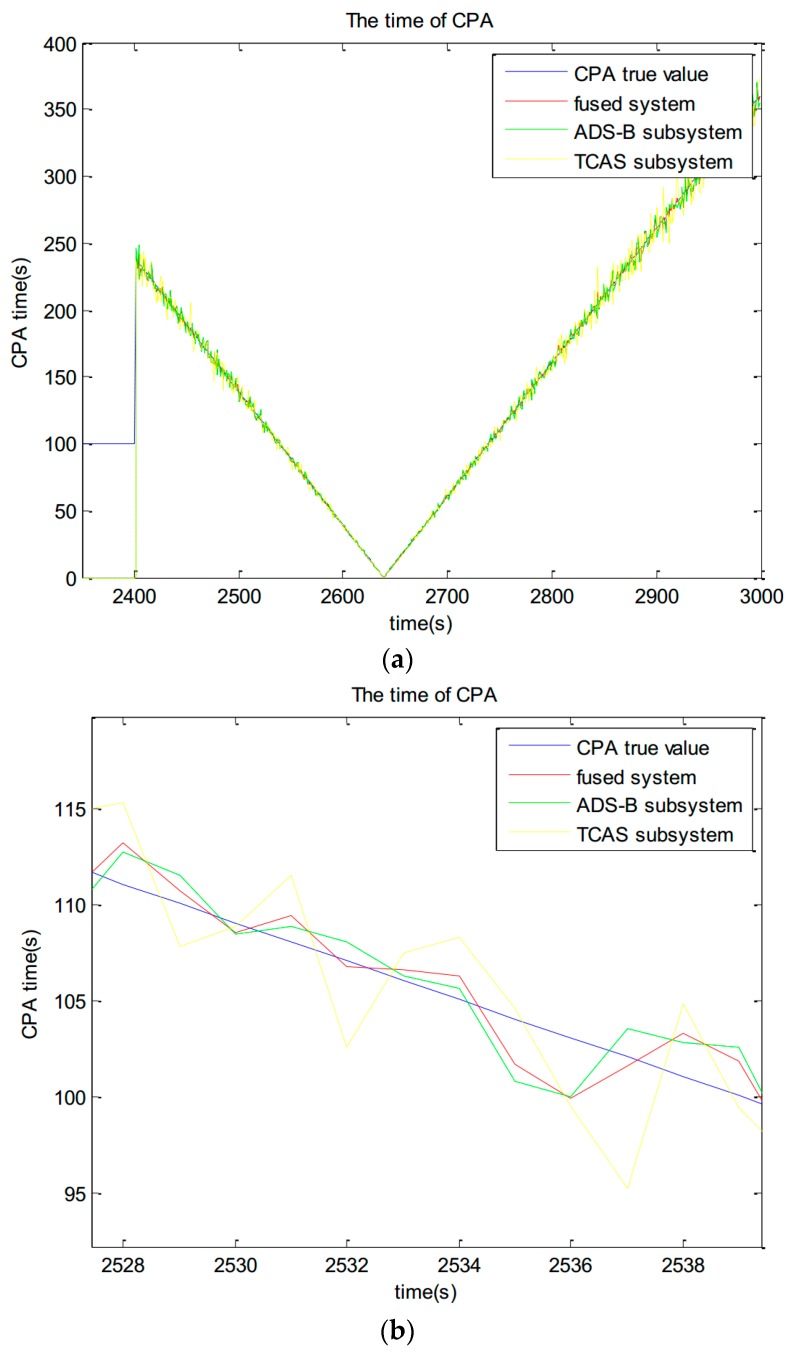
The CPA calculation results. (**a**) The CPA calculation results in large time period; (**b**) The CPA calculation results in small time period.

The CPA calculation results in large time period are shown in Figure 11a. The CPA calculation results in small time period are shown in Figure 11b. A fused track is added to the TCAS core solver model [30] to calculate the time of CPA. The trajectory is filtered by a VSVB-IMM model and the results of the fusion model are injected into the TCAS logic. The red line which represents the fused system is more close to the blue line which represents the CPA true value than any other subsystem.

**Figure 12:**
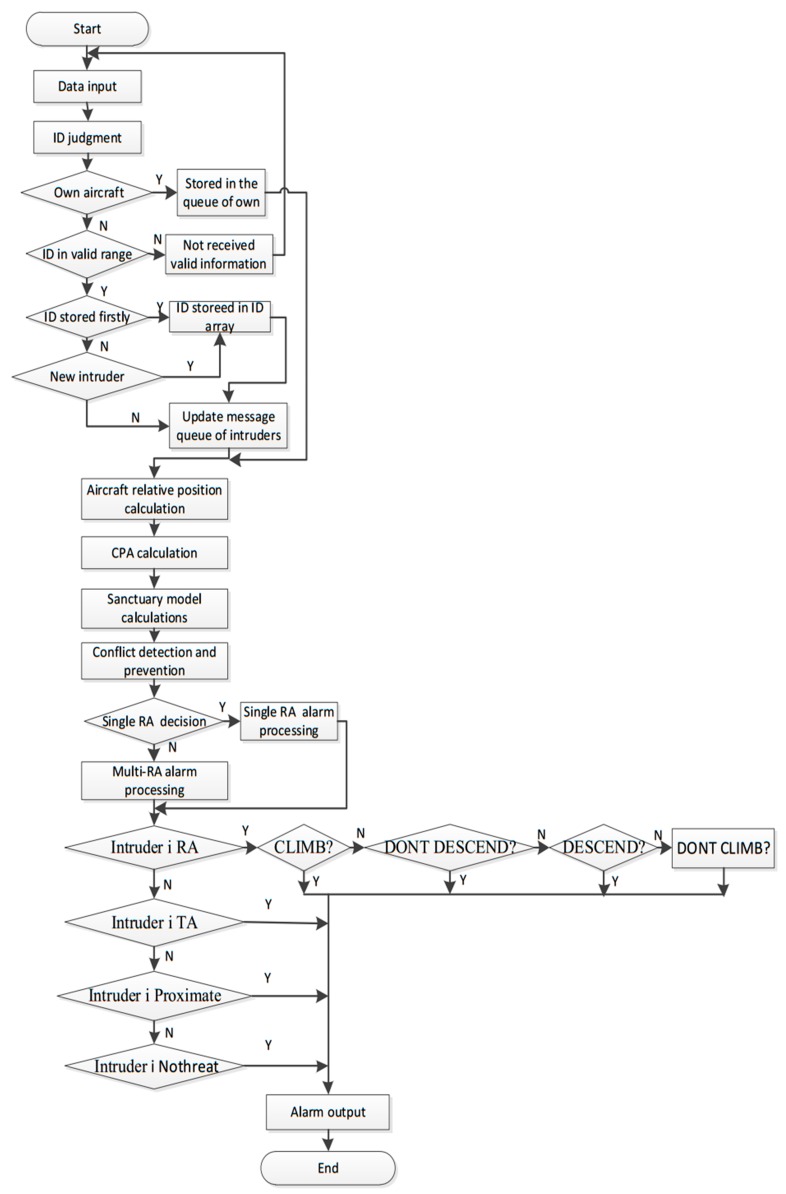
TCAS algorithm flowchart.

Figure 12 is a flowchart of the TCAS algorithm. This algorithm firstly generates track information by a flying solver model. The core processing program then receives data, conducts geodetic coordinate system and coordinate system conversion, executes the CPA algorithms and calculates the relative position of aircraft. Finally, it is vital to estimate the time of encounter, anticipate conflicts and make RA decisions. The relative position of aircraft is calculated and the value of Tau is estimated based on trend extrapolation method. TA is the Traffic Advisories and RA is the Resolution Advisories in TCAS.

5. False alarm, leak alarm analysis of fused system

**Figure 13:**
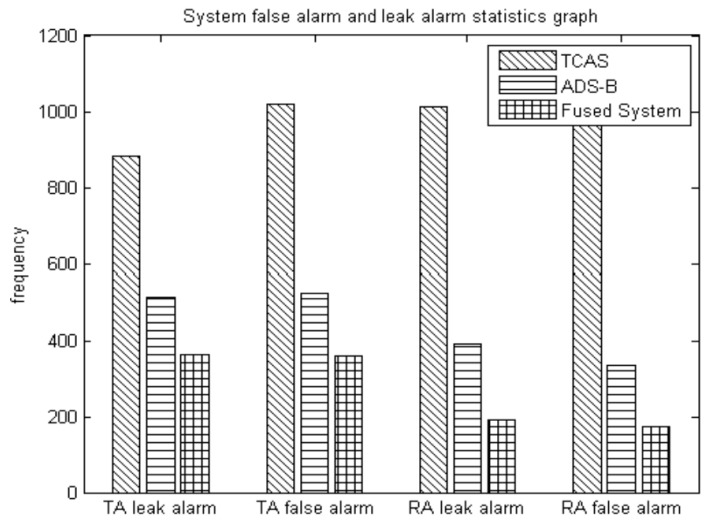
System false alarm and leak alarm statistics graph.

The experiment is repeated for 200 times independently. One of the statistics is the number of false alarms and leak alarms during the TA (CPA in 35–45 s) and RA (CPA < 35 s) period. Statistics for false alarms are when the actual time is ahead of the theoretical alarm time and exceeds the threshold value (it can be set to a constant 1 s). A leak alarm is when the actual time hysteresis of the theoretical alarm time exceeds the threshold value (1 s). In conjunction with Figure 13 and Table 2, qualitative and quantitative analysis can be carried out showing that the fused system can reduce the incidence of false and leak alarms in the TA and RA warning alarm interval. Leak alarms and delayed alarms compress the avoidance response’s time of the system and pilot, thus seriously affecting flight safety. Therefore, a more accurate alarm time can improve system security.

6. CPA cumulative deviation analysis after RA decision

**Figure 14:**
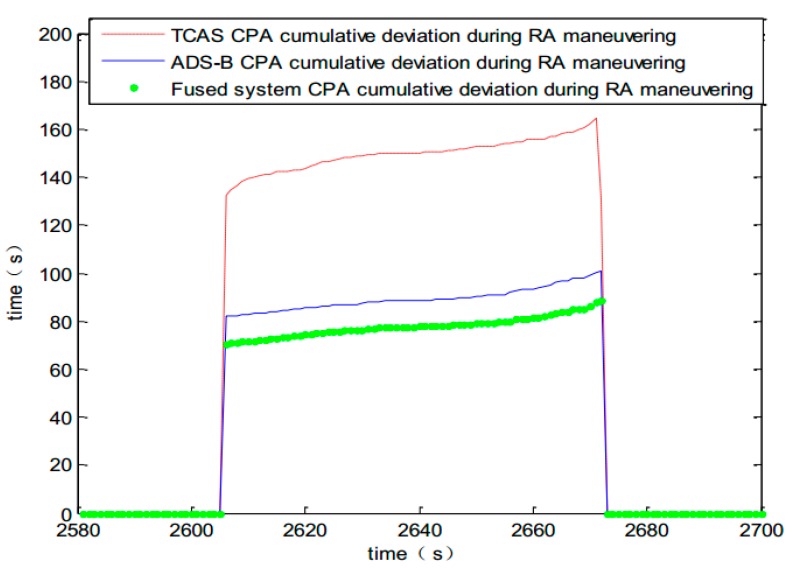
Accumulated deviation of CPA during the RA decision interval.

**Figure 15:**
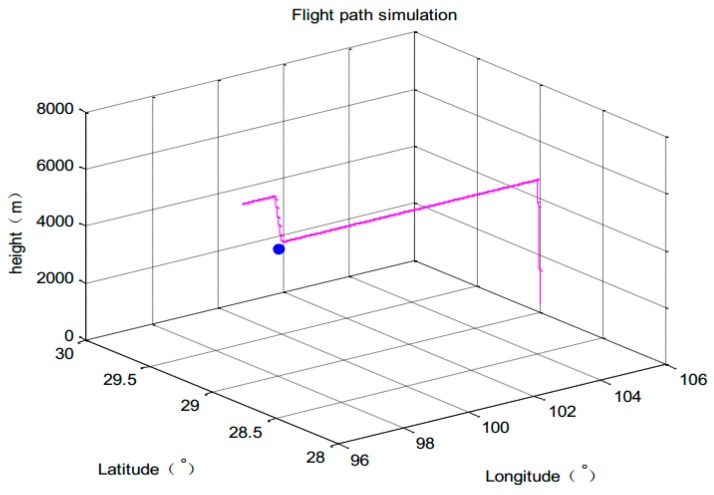
Track graph of simulating maneuvering avoidance during RA.

The TCAS core processor assesses the situation of the neighboring airspace and makes RA decisions. The intruder climbs to avoid detection at rate of 1500 feet per minute and meantime simulates the dynamic response where a human is in the loop. Pilot’s delay of practical response, mechanical and electrical system’s response delays can be taken into account. The delay was assumed to be a constant in this paper during the computer simulation. The simulation accumulated deviation data during the RA maneuver interval. It can be drawn from Figure 14 that the fused system can improve the cumulative deviation of CPA, which ensures a more precise response to ensure flight safety. Track graph of simulating maneuvering avoidance during RA is shown in Figure 15.

##### Application of ADS-B and TCAS Integrated Display

This part describes the engineering experiments and implementation of the ADS-B and TCAS data fusion algorithm. ADS-B message decoding, which obtains real-time flight information of the aircraft, is an important part of the ADS-B In. The main process of ADS-B message analysis is that ADS-B station or airborne equipment receives an ADS-B message and sends messages to the PC terminal through the network port. Then the message is decoded in the PC.

The data processing which includes calling the decoding function to decode the message, obtaining the message type and storing the corresponding variable is done. The message type has the air position information message (longitude, latitude and altitude in the air), the location information message (the longitude, the latitude, the running speed and the running speed direction of the ground target), the flight number information message (ICAO three word code, flight class set and flight category), speed information message (ground speed, ground speed and vertical speed) and error data messages.

Different ADS-B message types have different data composition order and data types. ADS-B message decoding program is that each data for independent analysis firstly and then the data analysis results are integrated, and finally the data statistics are calculated and stored.

**Figure 16:**
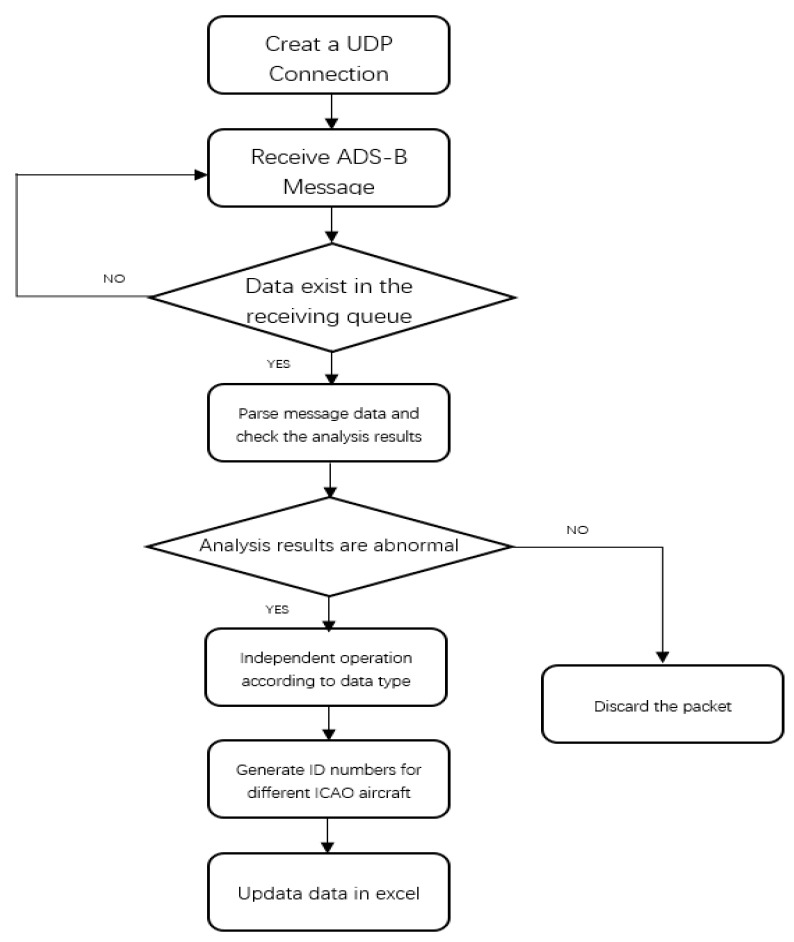
ADS-B message decoding software flow chart.

The ADS-B message decoding program records the aircraft information received from the airspace. For each aircraft, the program updates the information and all of information is written in a file when a new ADS-B message is received and decoded. Table 3 is part of the ADS-B ground station to receive part of the aircraft information, receiving a location in the latitude 31°01′31.79″ N east longitude 121°26′29.75″ E near the range of about 300 km. The flow chart of the ADS-B message decoding software is shown in Figure 16.

The message decoding software process mainly includes three independent work modules: message reception, message decoding, processing and saving. An ADS-B message is transmitted in the link of UDP with a point-to-point protocol. The process of message analysis is shown in Figure 16 and the results of message processing and saving are shown in Table 3, which contains the discarding process for abnormal packets.

The integrated display of ADS-B and TCAS is based on message analysis. The ADS-B message received from the airspace by the ADS-B equipment is decoded by the PC terminal. The message is transmitted to the fusion system as an input in the simulator cockpit platform. The purpose of system design is to implement some functions including functionally testing the TCAS subsystem, improving the accuracy of TCAS prediction, reducing unnecessary alarms, and improving the effectiveness and safety of the system. The interface of the fusion system (TCAS Digital Mock-Up) in the laboratory is shown in Figure 16. If there is a possibility of aircraft traffic collisions happening, the alarm status will appear in the area.

**Figure 17:**
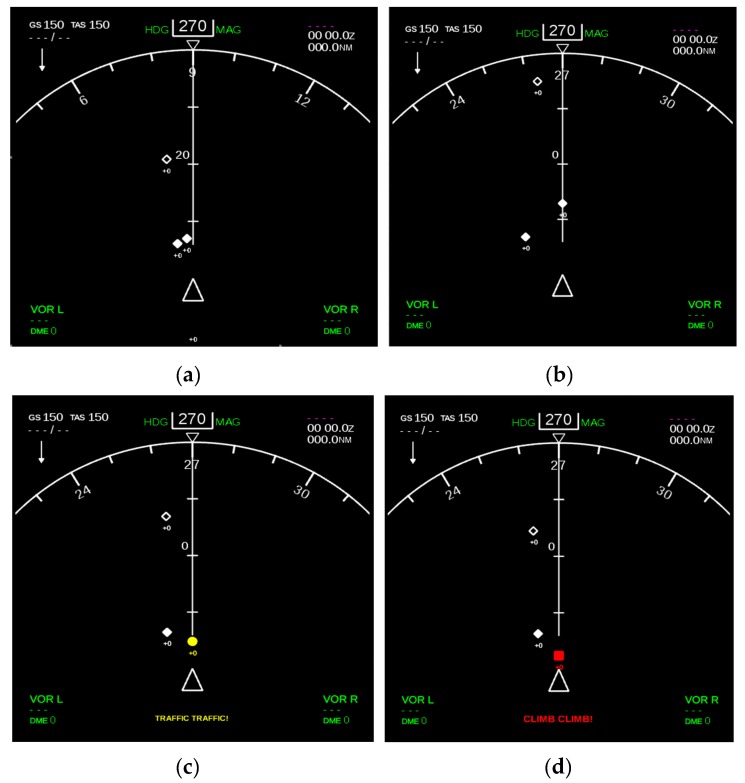
TCAS Digital Mock-Up. (**a**) Nothreat; (**b**) Proximate; (**c**) TA; (**d**) RA.

TCAS digital Mock-Up, which is the platform of ADS-B and TCAS fusion system, can also handle single or multiple intrusion aircrafts including the four states of No threat, Proximate, TA and RA. The conditions for the system to work normally are that the integrated exciter software, integrated digital Mock-Up software and communication of all modules is normal. It combines the fusion model based on VSVB-IMM into the core algorithm which has been described above. The input of the fusion system (TCAS Digital Mock-Up) can be real-time flight data generated by an aircraft in the airspace or simulated flight data from the cockpit platform. The four situations including Nothreat, Proximate, TA and RA are shown in Figure 17a–d.

#### 3.1.2. Display Interface Development

**Figure 18:**
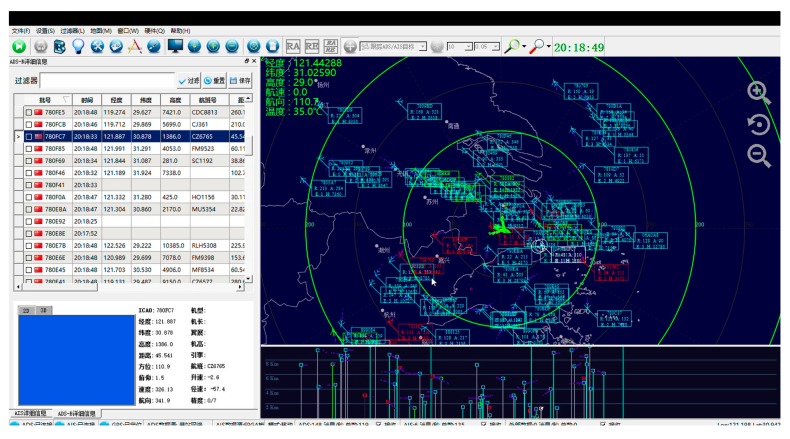
Airspace situation interface 1.

Figure 18 shows the airspace situation interface, which is able to display traffic conditions around 300 km in the air. The flight information which includes longitude, latitude, height, ground speed, ground speed direction, vertical speed and the type of aircraft is obtained by clicking on an aircraft icon. The flight path and height track information can also be more convenient to read out through the interface.

### 3.2. ADS-B Out Minimum System Implementation

#### 3.2.1. ADS-B Out Data Transmission Based On Simulation Cockpit Platform

**Figure 19:**
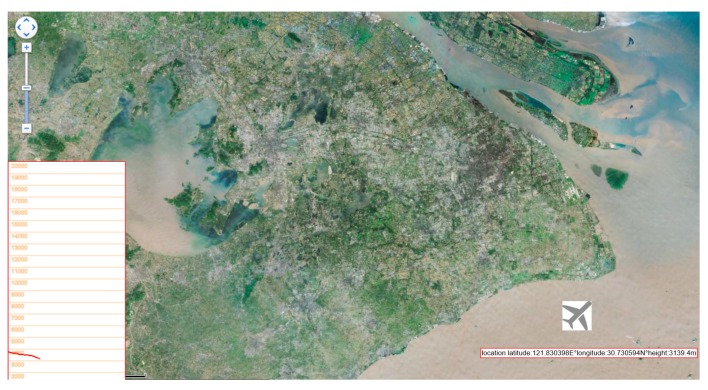
Airspace situation interface 2.

Simulated flight information which is generated by the simulation cockpit platform is transmitted to the airspace to simulate a real aircraft signal. Then the signal is received by the ADS-B station. Figure 19 shows the position of simulated aircraft and the signal received from the ADS-B station in the laboratory. The position of simulated aircraft is near the Shanghai Pudong Airport and over the airspace of the East China Sea. The process has benefits for experimental simulation work including traffic collision avoidance simulation, flight trajectory surveillance and verification, etc.

The airspace situation interface 2 is a new airspace situation interface in order to show some of the geographic information details. The interface can be modified to display 2D maps, satellite maps, or both by clicking on an icon. A variety of information including height trajectory of aircraft and other flight information is presented in it. The interface also supports two forms as input which are real-time flight data display and reads the saved data.

The development of the interface used a variety of Baidu map APIs to support the click control, slide the mouse wheel map zoom, click on the map switch, display the monitored aircraft location information and display the monitored aircraft height trajectory information. The interface implementation process is divided into four parts: create a map, set the map events, add controls to the map and add a cover to the map.

Compared with the monitoring interface shown in Figure 18, the airspace situation interface of Figure 19 which can be switched between 2D maps, satellite maps and mixed maps with clearer geographical features that can show more details of the map. The movement of the aircraft also shows a more intuitive and follow-up scene surveillance so research can continue on this basis.

#### 3.2.2. ADS-B Out Data Transmit Based On UAV Platform

**Figure 20:**
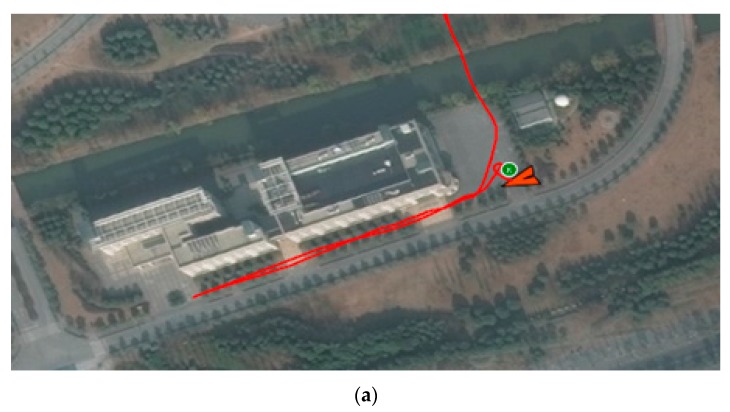

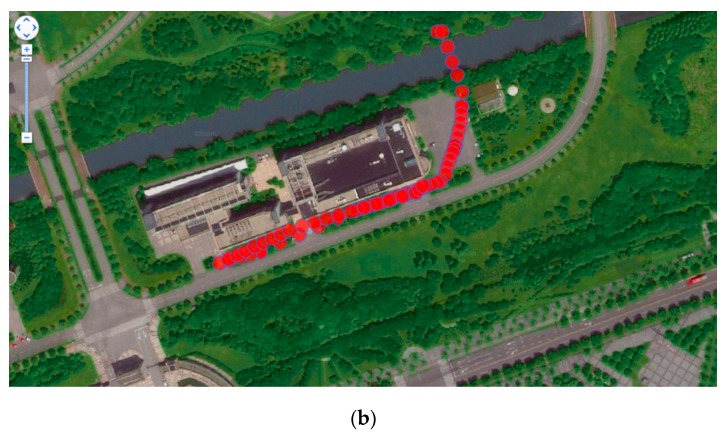
Unmanned Aerial Vehicle (UAV) trajectory verification. (**a**) Flight trajectory with software on the UAV; (**b**) Flight trajectory from ADS-B signal.

In this experiment, ADS-B airborne equipment was installed on the UAV platform to test ADS-B data transmission. The flight information is transmitted out by ADS-B airborne equipment and then received by an ADS-B station. Figure 20 is the flight trajectory with software on the UAV and the ADS-B signal.

By comparison, the ADS-B signal emitted by the ADS-B airborne equipment mounted on the UAV platform is consistent with the recorded results of the UAV’s own GPS flight track recorder. The experimental results show that the GPS module on the ADS-B airborne transmitter has a certain position accuracy and can meet the basic flight test and test requirements of the UAV platform.

## 4. Conclusions

Safeguarding human safety during aviation activities is the goal pursued by aviation practitioners and researchers. In our prior study, the simulation deviation of the fusion system is small but it is not sufficient to prove the effectiveness of the fusion benefit, so this paper built a minimal system based on an ADS-B physical system and TCAS simulation combined.

Based on the theory of ADS-B and TCAS, the paper designed and implemented an ADS-B minimum system from both theoretical and engineering aspects. A fusion model with a variable sampling VB-IMM (VSVB-IMM) algorithm is proposed in the theoretical aspect. A series of experimental simulations are done to verify the benefits of the fusion system including higher precision, lower false alarm rate and better flight security. In the engineering aspect, an airspace traffic situation display, ADS-B Out data transmission based on a simulation cockpit platform and an UAV platform are designed and implemented from software architecture and comparative analysis design of the results.

More in-depth studies can be done in the future on the benefits of fusion system security and large data analysis of airspace surveillance. In time synchronization, some methods will be added to consider the measured time which is from the GPS navigation unit to transponder. The ADS-B and TCAS fusion system would be improved by exploring two-system fusion methods which meet the benefits and security of ADS-B and TCAS fusion. Pattern recognition or other methods can be used to identify the model which makes it possible to have traffic collision situations in the surveillance airspace and to alarm or perform some other action.

## Figures and Tables

**Table 1:** Simulation sampling period corresponding to different Navigation Accuracy Category for Position (${\mathrm{NAC}}_{P}$ ) values.

$NAC_{P}$	Horizontal Accuracy Bound	Simulation Sampling Period
0	EPU ≥ 18.52 km (10 nm)	1 s
1	EPU < 18.52 km (10 nm)	1 s
2	EPU < 7.408 km (4 nm)	1 s
3	EPU < 7.408 km (4 nm)	1 s
4	EPU < 1852 m (1 nm)	1 s
5	EPU < 926 m (0.5 nm)	0.8 s
6	EPU < 926 m (0.5 nm)	0.6 s
7	EPU < 185.2 m (0.1 nm)	0.6 s
8	EPU < 92.6 m (0.05 nm)	0.6 s
9	EPU < 30 m	0.8 s
10	EPU < 10 m	1 s
11	EPU < 3 m	1 s

**Table 2:** Statistics of early alarm and hysteresis alarm during ta (35–45 s), ra (<35 s) in 200 experiments.

Alarm Type	System Categories		
	TCAS	ADS-B	Fused System
False alarm (TA) (frequency)	1018	524	361
Leak alarm (TA) (frequency)	883	513	362
False alarm (RA) (frequency)	1104	334	172
Leak alarm (RA) (frequency)	1014	390	192

**Table 3:** Part of aircraft information.

ID	Latitude	Longitude	Altitude	Speed North	Speed West	Speed Vertical
1	31.3268	122.629	4236.72	−45.2266	−312.967	−1728
2	31.3854	122.902	6156.96	−80.2507	−364.945	−1216
3	30.4038	121.241	4899.66	321.002	34.9823	64
4	30.6787	121.279	4038.6	351.002	36.9813	−1408
5	30.1474	121.154	5212.08	308.041	163.924	0
6	31.0127	122.763	7734.3	26.3675	477.98	1408
7	31.4885	123.445	7467.6	−76.2423	−351.948	64
8	29.8201	120.952	6454.14	355.048	191.911	−192
9	31.4381	123.173	7132.32	−82.2482	−362.944	−1088
10	31.0003	122.692	6156.96	0.335254	421	1792
11	30.2619	121.207	2834.64	263.014	87.9569	1088
12	31.7112	119.996	5394.96	−369.053	205.905	−2880
13	29.9003	121.456	3916.68	−13.2618	−336.99	1664
14	29.7201	122.295	8648.7	−94.3169	456.935	64
15	31.3854	122.902	6156.96	−80.2507	−364.945	−1216
